# Maturity Status and Relative Age of Elite Taekwondo Youth Competitors—Case Study on Croatian National Team

**DOI:** 10.3390/sports12020062

**Published:** 2024-02-19

**Authors:** Ana Kezic, Matej Babic, Drazen Cular

**Affiliations:** 1 Faculty of Kinesiology, University of Split, 21000 Split, Croatia; matej.babic@kifst.eu (M.B.); drazen.cular@kifst.eu (D.C.); 2 Faculty of Kinesiology, University of Zagreb, 10000 Zagreb, Croatia; 3 European Institute for Talents, Education, Research & Development, 21000 Split, Croatia

**Keywords:** biological age, peak of height velocity, talent identification, body composition, RAE, constituent year effect

## Abstract

This study examines the maturity status and relative age effect in elite youth taekwondo Croatian National Team athletes. Measurements of biological age, maturity offset, and body composition were taken from a sample of 17 junior athletes. Differences in maturity status were observed among athletes of the same chronological age, with variations in sitting height and age at peak height velocity. Male athletes generally exhibited higher values in body height, percentage of body fat, muscle mass, and total body water. No significant relative age effect was found. These findings highlight the importance of considering individual biological age and maturity status for talent development and training program adjustments. Further research involving athletes from different countries is recommended to validate these results and enhance the understanding of youth taekwondo athlete development.

## 1. Introduction

According to the official World Taekwondo Federation, taekwondo is practiced in more than 200 countries and five different continents. It is one of the most popular kicking combat sports, given the use of polystructural activities with open and semi-open movement structures [1]. Youth taekwondo competitions are organized to cater to younger practitioners, providing them with a platform to showcase their skills and compete in a controlled environment. Youth athletes are often grouped into weight categories (mostly seven female and seven male categories) to ensure matches are fair and balanced. This prevents significant size and weight differences between competitors. The theory of the specific physical characteristics (morphology) of taekwondo athletes is associated with optimal performance and it differs in different age categories, as well as weight categories. The researchers state that the selection of taekwondo elite athletes should rely on sport-specific tailored physical tests, integrating the relative information with that gathered from physiological measurements [2]. Although it is crucial to test these characteristics, research has shown that medalist and non-medalist elite taekwondo athletes in the European championships do not differ in terms of anthropometric and body-composition components and have a similar profile [3]. As with previous ones, other studies have also investigated body composition in elite taekwondo athletes [4,5,6,7], but only a few of them were conducted based on weight categories [8,9,10]. Research that permits information about the body composition of top athletes solely based on chronological age does not provide a thorough insight into the actual situation in different weight categories and, thus, prevents the adjustments of training programs for each category individually. Moreover, serious research should include elite athletes who are the best in their countries and who compete in the world’s greatest competitions.

Puberty is a period in which dramatic changes occur in the tempo and the timing of a growth spurt for an individual young athlete. Chronological (calendar) age and biological age can be very different, and biological age seems more appropriate in assessing physical development. For example, the physical characteristics of two 15-year-old male athletes, the first with an early onset of puberty and the second who will reach puberty later, would probably be completely different, although they train and/or compete in the same “chronological” category. The difference in puberty growth can be increased even further by the “relative age effect” (RAE). The relative age effect is related to the birth date of individuals. Someone who was born on 1 January is almost a year older than someone who was born in the same year but on 31 December. Taken along with differences in puberty growth, the mismatch may be increased. The specific period in which the growth rate is at its peak is called the peak height velocity (PHV). The start of the growth spurt is called “the onset”. The onset takes place one year before the PHV. After the PHV, the growth rate slowly decreases until the child has finished growing. Determining the PHV is one way of mapping the child’s developmental age. The accelerated growth phase consists of two variables: (1) the timing (onset) and (2) the rate at which the growth acceleration takes place. The timing is when the growth spurt starts, and the pace is the speed at which the growth occurs. Each child has their own pace and timing, but trainers are often forced to work with early matures, intermediate matures, and late matures in the same group. By determining the PHV in advance, trainers can apply an individual approach, which is a practical and manageable way to compare young athletes’ characteristics. When athletes are allocated to age categories that contain several increments or years in the same age category, e.g., Youth Olympic Games (YOG) sports, the age span varies from 3 years. In these age bands, the effects of the relative age work over a longer period, leading to what is the RAE, also known as the constituent year effect (CYE) [11]. Given that the literature search yielded no results on CYE in taekwondo articles, we will use the term RAE in this paper. The RAE has been found in many sports and sports categories [11,12,13,14], but there are also sports where the RAE has not been identified [15] or in very few cases [16]. Many studies have found that the RAE mostly exists in male groups of athletes and much less in female groups, as well as a prevalence of RAE in younger categories [17]. Many solutions have been proposed to address the RAE in sport but most are theoretical and there has been no attempt to implement them [18]. Altogether, the RAE appears as a result of the talent identification and development structures, characterized by early selection and early specialization, and which consider performance as the prerequisite for gaining access to the next developmental stages.

Studies of the RAE in taekwondo are rare. Authors have mostly not found RAE relevant in this sport [16,19], except for one in Korea [20]. Some justify this with the weight categorization, while others with less interest in taekwondo sport in certain countries. However, some studies have identified the RAE in cadet categories [21]. This confirms the fact that the RAE is mostly present in younger categories, which could easily lead to mistakes in the selection of younger athletes.

Considering the possible large differences in weight categories and considering also the large individual physical differences between athletes themselves regarding maturity, PHV, and relative age effect, it is very important to present and analyze the state of elite junior athletes in taekwondo using the example of a group of juniors, members of the national team in taekwondo. Therefore, the main aim of this study was to determine the maturity status of male and female youth Croatian taekwondo national team members. Additional aims of this study were to identify the RAE’s presence, body composition, and possible differences between female and male subsample. Based on current research and the elite level of athletes, existence of the RAE is not expected. However, we expect to find differences between the subsamples as well as in early maturation for most athletes.

## 2. Materials and Methods

The subject sample used for this research comprised 17 members of the Croatian national taekwondo team members divided into two groups: male juniors (*N* = 10) and female juniors (*N* = 7). The members of the national team were chosen based on a ranking list made out of the results of three criteria tournaments, which also included the results at the National championships. The quality of the sample is further emphasized by the fact that the respondents won a total of 5 medals (gold = 3 and bronze = 2) at the Junior European Taekwondo Championships held in November 2021 (immediately after the results were collected for research purposes). The participants confirmed their identity with a taekwondo license (GAL). A precondition to participate in this research was a clean health status, which was verified by a medical certificate from a certified sports medicine physician. Before taking part in this study, the underaged participants provided written consent signed by their parent/guardian, i.e., a personal statement.

The variable sample was defined by the following: (a) identification variables (name and date of birth); (b) a set of morphological measurements: body height (cm), body sitting height (cm); and (c) a set of variables defining body composition: body mass (kg), body mass index, body fat (%), body fat (kg), muscle mass (kg), lean body mass (kg), and total body water (%). The body height variable was measured by using the Martin anthropometer (Holtain, Crymych, UK), with a scale precision of 0.01 cm, while the body sitting height variable was measured by using the Holtain Harpenden Sitting Height Table SKU: H98607VR (Holtain, Crymych, UK), with a scale precision of 0.5 cm. All other variables were analyzed by using the TANITA diagnostic scale (BC 780) (Tanita Corporation, Tokyo, Japan), i.e., a monitor that calculates body composition based on electrical resistance [22], which differs depending on the tissue through which the electrical energy is conducted (Bio Impedance Analysis). All measurements were taken in the morning, in the sports hall, following the predefined protocols that had been announced to the participants and their coaches in advance. Subjects did not consume a meal or ingest large amounts of fluid before the measurement, and refrained from physical activity or training and sauna 24 h before the measurement. Also, the subjects’ bladders were emptied before performing the measurement. Hand–body and –thigh contact was avoided during the bioimpedance measurements. The measurers who performed the measuring were all experts in the field. Some factors limit the valid application of bioelectrical impedance analysis but only in extreme conditions, like obesity [23], whereas measurements validated for specific ethnic groups, populations, and conditions (which is the case in this study) can accurately measure body fat in those instances [24]. This study was conducted as a part of the following project: Biological, chronological and relative age in the function of developing Croatian national sport talent system, supported by the Croatian Science Foundation No. 3366 and the Ethical Board of the Faculty of Kinesiology University of Split, Croatia approved the described protocol (permit number: 2181-205-02-05-20-006, date: 26 February 2020). Calculation of the growth spurt was based on the following data: (a) date of birth, (b) test date, (c) gender, (d) body height, (e) sitting height, and (f) body weight. Research by Mirwald et al. [25] has determined that measuring the maturity offset (age at PHV) using all the variables previously discussed provides a valid and reliable prediction of the maturity status. These parameters are translated using a mathematical formula to the age at which the child is at the top of the growth spurt. This age is called the age at peak height velocity (APHV) and represents the number of years before or after the peak height velocity (PHV). The phases are as follows: 1. The pre-adolescent period (P1): This period starts a year before the start of the accelerated length growth (the onset). 2. The adolescent period (P2): Boys: from the onset to an average of three months after the APHV. So, 0.2–0.4 years after the APHV = total of 1.3 years. Girls: from the onset to an average of six months after the APHV. So, 0.3–0.9 years after the APHV = total of 1.6 years. 3. The post-adolescent period (P3): Boys: from two to four months after the APHV to 1.7 years later. Girls: from three to nine months after the APHV to 1.4 years later.

Data analysis methods included the calculation of descriptive statistical parameters: mean values, minimum result (minimum), and maximum result (maximum) for all variables, separately for males and females. Analysis of variance was used to determine the gender differences. The Chi-square test was used to identify the existence of RAE in this sample of athletes. For all statistical analyses, type one error was set at α = 5%. All calculations were performed by using the Statistica v.13 data analysis software system (TIBCO Software Inc., Palo Alto, CA, USA).

## 3. Results

The best Croatian junior taekwondo athletes of both sexes, members of the Croatian national team, were tested for this research and their results are presented in the tables below. Table 1 comprises the results of biological age parameters as well as maturity offset for each athlete (weight category), both male and female. Descriptive parameters have been calculated for both groups of athletes (mean value, minimal, and maximal result). Also, the last row of the table presents the results of ANOVA between the two groups. According to the p-values, male and female athletes differ significantly in the variable of sitting height (*p* = 0.02) in favor of male athletes, and in the variable of age at PHV (*p* = 0.00) in favor of male athletes. Numerically, they differ in the maturity offset as well; however, the difference was not significant. Regarding the maturity phase, female athletes are all in the third maturity phase already, while some of the male athletes, especially those in lower weight categories, are still in the second phase.

The results of the body composition variables for each athlete, measured by the TANITA scale, are presented in Table 2, as well as their average values, minimal, and maximal results for each group. Analysis of variance confirmed significant differences in body height (*p* = 0.03), percentage of body fat (*p* = 0.02), muscle mass (0.05), and total body water (*p* = 0.01), all of which were in favor of male athletes, except for the percentage of body fat, which was significantly higher in female athletes.

**Figure 1:**
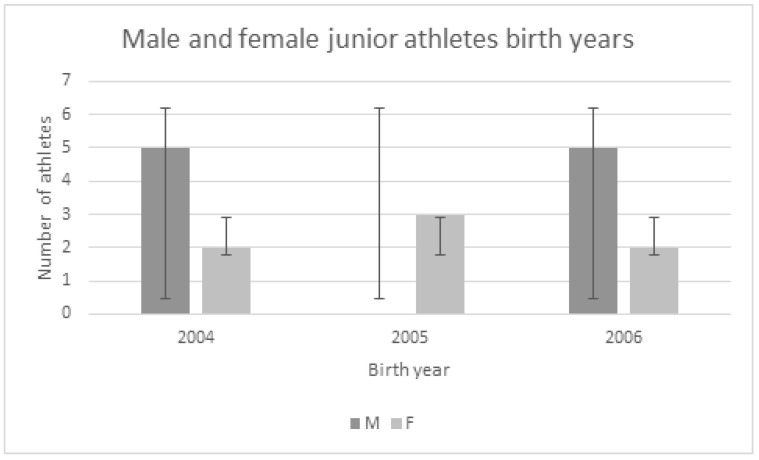
Relative age of elite male and female junior taekwondo athletes.

All the athletes were grouped according to their birth year for RAE analysis, and the results are presented in Figure 1. In male junior athletes, the analysis of frequencies did not determine the existence of significant differences between the observed and expected frequencies according to birth year (Chi-square test), with a *p*-value of 0.17. The largest number of successful male junior taekwondo athletes were born in the first- and last-possible birth year.

When observing the female sample of junior taekwondo athletes, similar to the male sample, Chi-square analysis did not determine significant differences between the observed and expected frequencies according to birth year, with a *p*-value of 0.96. It also needs to be noted that the largest number of female taekwondo athletes were born in the middle-most birth year possible for junior athletes.

## 4. Discussion

The present study analyzed the maturity status, RAE, and body composition of male and female junior Croatian taekwondo national team members. The initial idea was to test the best junior athletes in the country, as they can serve as a model for possible talent identification, and check their biological age and the possible influence of relative age in their success. In this matter, we included only the best athlete in each weight category, and their results are presented directly.

The analysis shows that although the athletes are on average of the same chronological age, their biological age differs. This is obviously due to different maturity offset numbers, meaning that the number of years the athlete is away from the PHV is different for each individual even though they are in the same age category. Further, the maturity phase variable in male athletes showed that some of them are still in the second maturity phase, somewhat behind their peers, and still managed to reach the national team in their weight category. These athletes should be monitored and coached carefully because following their further maturation, additional progress in performance is expected. This is more important because the processes of physical growth, biological maturation, and behavioral development occur simultaneously, interact with each other, and are a crucial matter for talent development [26]. Based on this, researchers have been identifying the need for academics and practitioners to work more closely to establish an evidence base related to accelerated and decelerated periods of athletic development during maturation [27].

When comparing the results between male and female athletes, they differ significantly in the variable of sitting height and age at PHV in favor of male athletes. The differences in age at PHV specifically confirm the fact that females mature earlier than males [28]; therefore, they can expect to reach certain motor-functional potentials sooner. This is probably the reason why female athletes achieve success at a significantly younger age than their male counterparts. Regarding the age of athletes in different weight categories, the same authors reported that female athletes in the flyweight category were younger than those in the heavyweight category, and that male athletes in the flyweight and featherweight categories were younger than those in the welterweight and heavyweight categories. This situation can be confirmed in the male sample of athletes in this study also, whereas male junior athletes in lighter categories tend to be younger. However, the same is not the case in the female junior national team.

In addition to differences in maturity levels between junior male and female athletes, the analysis shows differences in body composition variables as well. Significant differences in body height, percentage of body fat, and muscle mass, but not in body mass index, is a phenomenon that has been confirmed in some previous research of taekwondo athletes [29,30]. When compared to senior elite taekwondo athletes [29], the Croatian national junior team has lower values of BMI, both male and female. Therefore, body composition variables could serve as an auxiliary factor in determining the quality of taekwondo athletes, but certainly not as a decisive factor, based on previous research [3]. What could also be helpful in talent recognition of elite taekwondo athletes is the session-RPE (subjective ratings of perceived exertion) and physiological measurements [2].

Regarding the presence of the RAE in the sample of elite junior taekwondo athletes in Croatia, it was not identified in either male or female categories. This was somewhat expected since previous authors have not found the RAE in their investigations on taekwondo athletes [16,19]. On the other hand, through meta-analysis Albuquerque et al. [31] have identified that the RAE was significantly higher in male categories compared to females. Also, most of the research has been executed in the senior category and it was possible that there could have been a different situation when junior athletes were involved. Korean scientists found that athletes with early birth months had relatively high winning results in national competitions [20]. It has to be emphasized that this research was performed on thousands of athletes of all ages and it did not specify the age category, so it would be difficult to compare these results with those of this study and draw specific conclusions. Babic et al. [21] have found the presence of the RAE in Croatian cadet taekwondo athletes, which confirms the fact that the RAE can be more present in younger categories [17]. However, this did not happen in a national junior team where most males were born in the first- and third-possible birth year. The analysis for the female sample also did not confirm the presence of the RAE, whereas most of them were born in the middle-most birth year possible for junior athletes. This is once more a confirmation that the RAE is not a significant factor in junior taekwondo athletes’ success and that far more valuable information can be obtained through biological age detection and maturation analysis. Since it was noted that determining biological age is crucial for talent development [26], such procedures need to undoubtedly be implemented into all sports preparation procedures, especially at the national team level. Only in this way can we claim that the system fully supports the development of sports talents, respecting the multidisciplinary approach to talent development, which is often neglected [32]. In addition to biological age, the variable of training age or previous training experience can serve to explain in more detail what kind of experience and background elite athletes continue to practice taekwondo with, mostly regarding early athletic education and early technique learning. So, these should also be incorporated into talent detection and development phases.

This study provides valuable insights into the role of biological age, maturity phase, body composition, and relative age effect in athletic performance. The significant differences observed between male and female athletes in terms of sitting height, age at PHV, and body composition variables highlight the importance of considering sex-specific factors in athlete development. The lack of a significant RAE in this sample suggests that other factors, such as weight categories, biological age, and maturity status, may be more influential in determining success in taekwondo.

Moreover, the results emphasize the importance of monitoring and coaching athletes carefully, particularly those who are still in the earlier stages of maturation. As these athletes continue to mature, they are likely to experience further progress in performance. Therefore, implementing procedures to determine biological age in sports preparation, especially at the national team level, is essential for supporting the development of sports talents. Finally, while the current study provides valuable insights, further research is needed to explore the complex interactions between these factors and how they influence performance in taekwondo and other sports. Future studies could also investigate the potential impact of other physiological aspects on athletic performance.

## 5. Conclusions

To address the challenges posed by RAE and CYE in individual sports like taekwondo, we propose several tailored approaches for practical application in taekwondo sport:

(a) Tailored age categorization in taekwondo:

Unlike team sports, taekwondo involves weight and recently also height categories (in cadet age categories), which already provide a form of categorization. However, integrating age categorization within these weight/height divisions can further refine the selection process, leading to fairer competition and more accurate talent identification, essential for nurturing young taekwondo athletes.

(b) Chronological and biological age integration:

The integration of chronological age with biological maturity assessments in taekwondo can offer a more comprehensive understanding of an athlete’s capabilities. This can involve adjusting evaluations or rankings based on an athlete’s relative age and biological maturity. Such a strategy can help coaches and selectors recognize and support talents who might be at a physical disadvantage due to later development, ensuring equitable opportunities for all athletes within the same chronological age group.

(c) Emphasis on biological age for talent identification:

Incorporating biological age measurements, such as maturity offset or bone age, into the talent identification process in taekwondo can be crucial. This approach allows for a more nuanced understanding of an athlete’s developmental stage, ensuring training and competition categories are aligned with their physical maturity. This is particularly important in a sport like taekwondo, where physical attributes such as strength, flexibility, and reach are pivotal.

## Figures and Tables

**Table 1:** Maturity offset and biological age parameters of elite youth taekwondo athletes, separately for male and female athletes; results of analysis of variance between the groups.

Male							
	WC	DoB	CA	SBH	Age@PHV	MO	MF
Subject 1	−45 kg	17 June 2006	15.4	87.1	14.5	0.9	P2
Subject 2	−48 kg	28 October 2006	15.0	86.3	14.3	0.7	P2
Subject 3	−51 kg	7 November 2006	15.0	90.9	13.7	1.3	P2
Subject 4	−55 kg	14 December 2004	16.9	89.5	14.8	2.0	P3
Subject 5	−59 kg	15 March 2006	15.6	93.4	13.6	2.0	P3
Subject 6	−63 kg	10 February 2004	17.7	94.7	14.3	3.4	P3
Subject 7	−68 kg	11 January 2006	15.8	93.3	13.7	2.1	P3
Subject 8	−73 kg	30 August 2004	17.2	98.6	13.4	3.7	P3
Subject 9	−78 kg	16 May 2004	17.5	97.9	13.6	3.8	P3
Subject 10	+78 kg	7 July 2004	17.3	104.8	12.4	4.9	P3
Minimum			15.0	87.1	13.4	0.7	P2
Mean value			16.34	93.65	13.8	2.4	
Maximum			17.7	104.8	14.8	4.9	P3
**Female**							
	**WC**	**DoB**	**CA**	**SBH**	**Age@PHV**	**MO**	**MF**
Subject 11	−42 kg	23 July 2006	15.3	85.7	12.9	2.3	P3
Subject 12	−46 kg	7 November 2004	17.0	86.8	13.3	3.6	P3
Subject 13	−49 kg	28 October 2004	17.0	89.0	13.0	4.0	P3
Subject 14	−59 kg	3 February 2006	15.7	85.3	13.0	2.8	P3
Subject 15	−63 kg	28 April 2005	16.5	91.6	12.4	4.1	P3
Subject 16	−68 kg	28 December 2005	15.8	85.9	12.7	3.2	P3
Subject 17	+68 kg	6 February 2005	16.7	90.9	12.7	4.0	P3
Minimum			15.3	85.7	12.9	2.3	P3
Mean value			16.3	87.9	12.9	3.4	
Maximum			17.0	90.9	13.3	4.1	P3
ANOVA (*p*-value)			0.90	0.02	0.00	0.12	

WC = weight category; DoB = date of birth (dd/mm/yy); CA = chronological age (year); SBH = sitting body height (cm); Age@PHV = age at peak of high velocity; MO = maturity offset (years); MF = maturity phase (P1/P2/P3).

**Table 2:** Reference body composition values of elite youth taekwondo athletes, separately for male and female athletes; results of analysis of variance between the groups.

Male									
	WC	BH	BM	BMI	BF%	BFkg	MM	LBM	TBW
Subject 1	−45 kg	169.9	46.5	16.10	7.5	3.5	40.8	2.20	68.8
Subject 2	−48 kg	170.0	50.9	17.60	11.40	5.80	42.80	2.30	65.6
Subject 3	−51 kg	172.4	52.9	17.80	15.40	8.10	42.50	2.30	62.6
Subject 4	−55 kg	178.5	55.7	17.50	11.70	6.50	46.70	2.50	64.5
Subject 5	−59 kg	173.0	60.4	20.20	9.50	5.70	51.90	2.80	66.4
Subject 6	−63 kg	178.6	66.4	20.80	10.00	6.60	56.80	3.00	62.8
Subject 7	−68 kg	187.9	68.5	19.40	8.90	6.10	59.30	3.10	64.4
Subject 8	−73 kg	190.6	75.5	20.80	13.70	10.30	62.00	3.20	58.3
Subject 9	−78 kg	191.4	81.9	22.30	8.20	6.70	71.50	3.70	59.8
Subject 10	+78 kg	195.6	89.6	23.10	15.10	13.30	71.40	3.70	54.8
Minimum		169.9	46.5	16.10	7.5	3.5	40.8	2.20	58.3
Mean value		180.8	64.8	19.56	11.14	7.26	54.57	2.88	62.8
Maximum		195.6	89.6	23.10	15.10	13.30	71.50	3.70	68.8
**Female**									
	**WC**	**BH**	**BM**	**BMI**	**BF%**	**BFkg**	**MM**	**LBM**	**TBW**
Subject 11	−42 kg	161.9	40.80	15.60	10.60	4.30	34.60	1.90	64.7
Subject 12	−46 kg	168.7	47.50	16.70	8.70	4.10	41.20	2.20	60.6
Subject 13	−49 kg	172.5	49.70	16.70	11.30	5.60	41.90	2.20	60.4
Subject 14	−59 kg	164.0	59.80	22.20	22.10	13.20	44.20	2.40	54.8
Subject 15	−63 kg	179.0	62.90	19.60	19.20	12.10	48.20	2.60	52.0
Subject 16	−68 kg	173.4	65.30	21.70	24.60	16.10	46.70	2.50	51.9
Subject 17	+68 kg	176.0	74.10	23.90	23.60	17.50	53.70	2.90	49.9
Minimum		161.9	40.80	15.60	8.7	4.10	34.60	1.90	49.9
Mean value		170.8	57.16	19.49	17.16	10.41	44.36	2.39	56.3
Maximum		179.0	74.10	23.90	24.60	17.50	53.70	2.90	64.7
ANOVA (*p*-value)		0.03	0.25	0.95	0.02	0.14	0.05	0.06	0.01

WC = weight category; BH = body height (cm), BM = body mass (kg); BMI = body mass index; BF% = body fat (%); BFkg = body fat (kg); MM = muscle mass (kg), LBM = lean body mass (kg); TBW = total body water (%).

## Data Availability

The data presented in this study are available upon request from the corresponding author.
